# Osteoporosis correlates with abnormal ocular vestibular evoked myogenic potential in patients with benign paroxysmal positional vertigo

**DOI:** 10.3389/fneur.2026.1785323

**Published:** 2026-03-12

**Authors:** Chen-Yan Zhou, Liang Shu, Jing Wu, Jie Chen, Ying-Xia Bai, Ran Yan, Xu-Hong Sun, Shuai Xu, Jian-Ren Liu, Hai-Bin Sheng, Wei Chen

**Affiliations:** 1 Department of Neurology, Shanghai Ninth People's Hospital, Shanghai Jiao Tong University School of Medicine, Shanghai, China; 2 Department of Neurology, Shanghai Second People's Hospital (Shanghai Huangpu District Central Hospital), Shanghai, China; 3 Department of Otolaryngology-Head and Neck Surgery, Shanghai Ninth People's Hospital, Shanghai Jiao Tong University School of Medicine, Shanghai, China; 4 Ear Institute, Shanghai Jiao Tong University School of Medicine, Shanghai, China; 5 Shanghai Key Laboratory of Translational Medicine on Ear and Nose Diseases, Shanghai, China

**Keywords:** benign paroxysmal positional vertigo, bone mineral density, osteoporosis, utricular function, vestibular evoked myogenic potential

## Abstract

**Background:**

Osteoporosis may increase the risk of benign paroxysmal positional vertigo (BPPV). However, direct evidence remains elusive.

**Aims:**

To analyze the correlation between bone mineral density (BMD) and vestibular function in elderly patients with BPPV.

**Methods:**

Two hundred ninety-one idiopathic, unilateral BPPV patients aged 50–80 years were consecutively enrolled in our vertigo outpatient clinic. All the participants underwent BMD, cervical, and ocular vestibular evoked myogenic potential (c/oVEMP) evaluations. The associations between BMD and VEMP results were investigated.

**Results:**

Eighty-one patients (27.8%) were diagnosed with osteoporosis, 120 patients (41.2%) had osteopenia, and 90 (30.9%) exhibited normal BMD. Among BPPV patients, abnormal BMD demonstrated a marginal correlation with oVEMP response (*p* = 0.098), but not with cVEMP response (*p* = 0.405). Compared to those without osteoporosis, patients with osteoporosis were older (65.9 vs. 62.7 years, *p* = 0.001), had lower BMI (22.6 vs. 24.3, *p* < 0.001), showed a higher proportion of females (84.0 vs. 72.4%, *p* = 0.039), and were more likely to present with at least unilateral oVEMP absence (74.1 vs. 57.1%, *p* = 0.008). Patients exhibiting at least unilateral oVEMP absence also had reduced T-scores and BMD in the lumbar spine. After adjusting for confounding variables, osteoporosis remained independently associated with at least unilateral oVEMP absence in BPPV patients (OR = 2.038, *p* = 0.019).

**Conclusion:**

Our study provides further evidence that osteoporosis may contribute to utricular dysfunction associated with the occurrence of BPPV.

## 1 Introduction

Benign paroxysmal positional vertigo (BPPV), the most common peripheral vestibular disorder, occurs when the otoconia detach from the utricle and enter the semicircular canal. Current evidence suggests that older adults (1), females (2), climate change (3), osteoporosis, vitamin D deficiency (4, 5), and cerebral small vessel disease are significant risk factors for BPPV (6). A large population-based Korean study involving about 350,000 participants reported BPPV incidence rates of 31.58 and 18.09 cases per 1,000 individuals in the osteoporosis and normal control groups, respectively, emphasizing that osteoporosis may be associated with increased occurrence rate of BPPV (7). The risk of BPPV recurrence is also elevated in people with osteoporosis (8). Moreover, the higher incidence of osteoporosis observed in BPPV patients vs. healthy controls indicates a potential association between the two conditions (9). Recent findings reveal the importance of vitamin D in maintaining inner ear stability, implying a physiological basis for the link between whole-body bone metabolism and vestibular disorders (10, 11). Nevertheless, direct evidence establishing a causal link between osteoporosis and BPPV remains insufficient.

Vestibular dysfunction is commonly observed in BPPV patients. Even after a successful canalith repositioning procedure (CRP), more than 30% of patients continue to experience dizziness or imbalance (12). Although positional tests (e.g., Dix–Hallpike) are standard for BPPV diagnosis, their sensitivity may be low outside the acute phase; therefore, complementary objective vestibular assessments can be valuable (13). Vestibular evoked myogenic potentials (VEMP) testing is a well-established and reliable electrophysiological method for evaluating vestibular responses at different segments of the vestibular pathway and assessing otolith organ function (14). Depending on electrode placement, VEMP recordings are mainly categorized into cervical and ocular types, referred to as cVEMP and oVEMP, respectively. Physiologically, the oVEMP reflects utricular function mediated by the vestibulo-ocular pathway, while the cVEMP provides an assessment of saccular function via the vestibulo-collic pathway. Previous studies have utilized changes in VEMP to evaluate vestibular dysfunction in BPPV patients (15, 16). BPPV patients have been reported to exhibit lower oVEMP amplitudes and greater interaural asymmetry ratios compared to healthy controls (17). Additionally, another study demonstrated that the cVEMP interaural amplitude difference ratio (IAD) was correlated with the prognosis of BPPV (18, 19). Our prior research demonstrated that evaluating cVEMP could help predict short-term residual dizziness following an effective BPPV repositioning procedure (20).

Research has indicated that low bone mineral density (BMD) is linked to balance impairments (21), and osteoporosis may contribute to vestibular dysfunction in the aging population, especially in postmenopausal women (22–24). However, it remains unclear whether abnormal BMD is correlated with vestibular dysfunction in patients with BPPV. We hypothesized that such a correlation may also be present in elderly BPPV patients. Furthermore, it is not yet established whether abnormal BMD is specifically associated with utricular or saccular dysfunction. This investigation focuses on exploring potential correlations between abnormal BMD and cVEMP or oVEMP outcomes in older individuals with unilateral idiopathic BPPV.

## 2 Materials and methods

### 2.1 Patients

In total, 291 BPPV patients suffering from this condition were enrolled in this research between June 2017 and February 2022. All BPPV patients complied with the diagnostic requirements of the Bárány Society (25): (i) Brief, recurrent episodes of dizziness or lightheadedness, typically lasting no more than 1 min, triggered by changes in head position relative to gravity. (ii) The positional test induced vertigo and revealed characteristic nystagmus. In posterior canal BPPV, the classic nystagmus is characterized by an upward-beating vertical motion with torsional components, observed when the affected ear is positioned downward during the Dix-Hallpike maneuver. In contrast, horizontal canal BPPV is identified by apogeotropic or geotropic nystagmus, which can be elicited during the roll test. (iii) Other conditions, such as Meniere's disease, orthostatic hypotension, labyrinthitis, psychogenic vertigo, vestibular paroxysmia, posterior circulation ischemia, and vestibular migraine, were excluded. This investigation employed the following inclusion standards: (i) Patients with a definitively diagnosed first-episode of unilateral BPPV, and the type of nystagmus was consistent with the involved semicircular canal. (ii) Patients aged 50–80 years. (iii) Have not taken calcium tablets in the past month. Participants were excluded based on the following criteria:(i) involvement of multiple semicircular canals. (ii) patients with superior semicircular canal BPPV. (iii) a history of trauma or other causes indicative of secondary BPPV. Each participant diagnosed with BPPV underwent successful repositioning maneuvers at enrollment. Therapeutic interventions were canal-specific: the Epley procedure was employed for posterior canal BPPV, whereas horizontal canal variants were managed with either the barbecue or Gufoni maneuvers (26). All participants completed both VEMP and BMD examinations within 1 week after CRP. This investigation was conducted with approval from the Ethics Committee of Shanghai Ninth People's Hospital Affiliated to Shanghai Jiao Tong University, School of Medicine (SH9H-2020-T270-2). All methods were performed in accordance with the relevant guidelines and regulations as stipulated in the Declaration of Helsinki. Each participant signed an informed consent document prior to enrollment.

### 2.2 Clinical profiles

The baseline data included sex, anthropometric data (height and weight), smoking habits, alcohol consumption history, and cardiovascular risk factors (notably hypertension, diabetes mellitus, and coronary heart disease). We evaluated the patients' symptoms at the first visit (W0) and 1 week later (W1). The severity of dizziness and vertigo was measured using a visual analog scale (VAS), which ranged from minimum (0 equals to symptom-free) to maximum (100 equals to peak severity). To successfully assess how vertigo affects patients' daily functioning, we utilized the Dizziness Handicap Inventory (DHI) questionnaire. The DHI is a widely used instrument for assessing vestibular symptoms and consists of 25 items, each with three response options: four points for “yes,” two points for “sometimes,” and 0 points for “no.” The inventory is divided into three subdomains: emotional (DHI-E), physical (DHI-P), and functional (DHI-F) scores (27). Patients' total scores fall within a 0–100 range, and more points signify negative effects on their everyday activities (28).

### 2.3 Bone mineral density evaluation

All enrolled individuals received baseline bone density evaluations within the study's first week. The evaluation was conducted using a X-ray absorptiometry (DXA) densitometer (America, Lunar IDXA), which is widely accepted as the most authoritative method for measuring BMD (29). The assessment included measurements at several key skeletal regions: the lumbar spine (L1–L4), greater trochanter, femoral shaft, Ward's triangle, and femoral neck. The T-score formula is as follows: (Patient's BMD—Reference population's peak BMD)/Reference population's peak BMD standard deviation. According to the WHO criteria, osteoporosis is diagnosed at ≤ −2.5, normal bone mass at a T score ≥ −1, and osteopenia at −2.5 < T score < −1 (9).

### 2.4 VEMP evaluation

VEMP evaluations were conducted in the hearing center of Shanghai Ninth People's Hospital, Affiliated to Shanghai Jiao Tong University School of Medicine. VEMP measurements were obtained using an auditory-evoked potential analyzer in a noise-controlled environment (Neuro-Audio, Neurosoft LLC, Ivanovo, Russia). Acoustic stimuli consisted of 500 Hz tone bursts delivered via IP-30 insert earphones at a rate of 5.1 repetitions per second, with averaged responses derived from 60 to 200 trials. Signal filtering was set with cutoff frequencies of 8 and 1,500 Hz. The recordings were deemed acceptable only when the electrode impedance did not exceed 5 kΩ.

During cVEMP assessments, participants were positioned supine with their heads rotated toward the side opposite to stimulation. Electromyographic activity was continuously recorded from the activated sternocleidomastoid muscle (SCM) during sound stimulation. Recording electrodes were placed on the proximal third of the SCM, with reference electrodes positioned at the sternal manubrium. A ground electrode was centrally positioned on the midline of the forehead. For oVEMP testing, participants remained supine and directed their gaze about 30 degrees above the head midline, a position known to elicit optimal responses. Recording electrodes were placed 10 mm below the midpoint of the infraorbital margin, with the reference electrode positioned an additional 10 mm below, while the ground electrode was placed at the central forehead. Throughout the examination, patients were instructed to maintain steady fixation on the target and minimize blinking. Non-responsiveness was defined as the absence of characteristic biphasic potentials, even at the maximum stimulation intensity (110 dB nHL) (20). In patients with bilateral presence of cVEMP or oVEMP, detailed parameters, including latency, thresholds, amplitude, and amplitude asymmetry ratio (AAR), were recorded. AAR was calculated as follows: 100 times (amplitude on right side—amplitude on left side)/(sum of right and left side amplitudes).

### 2.5 Statistical analysis

Data analysis was conducted in SPSS 26.0 for Windows (IBM Corp, Armonk, NY, United States), and figures were produced with GraphPad Prism version 10.0.0 (GraphPad Software, San Diego, CA, United States). The mean and standard deviation were used for normally distributed continuous variables, whereas the median and interquartile range (IQR, 25th to 75th percentiles) were applied to skewed distributions. *F*-tests and *T*-tests were conducted on normally distributed data, with the Mann-Whitney *U*-test applied to skewed data. Chi-square tests and Fisher's exact tests were used to analyze categorical variables. The independent correlates of abnormal oVEMP findings were examined through binary logistic regression analysis. Results with *p*-values below 0.05 were considered statistically significant.

## 3 Results

Among the cohort, average age of the patients was 63.6 ± 1.8 years, and 75.6% were female. 229 cases (78.7%) demonstrated posterior canal involvement, while horizontal canal BPPV was present in 62 individuals (21.3%). The involvement was right-sided in 185 (63.6%) patients and left-sided in 106 (36.4%) patients. Among the enrolled participants, 90 (30.9%) had normal BMD, 120 (41.2%) had osteopenia, and 81 (27.8%) had osteoporosis. According to the VEMP test results, 64 (22%) showed unilateral absence of cVEMP, 67 (23%) demonstrated bilateral absence of cVEMP, 83 (28.5%) exhibited unilateral absence of oVEMP, and 97 (33.3%) presented with bilateral absence of oVEMP. Meanwhile, the abnormal rate of c/oVEMP on the affected side of BPPV was similar to that on the unaffected side (35.7 vs. 32.3%, 45.7 vs. 49.4%, respectively). There was no difference in the abnormal rate of c/oVEMP between the affected and unaffected sides in the osteoporosis group with BPPV.

### 3.1 Characteristics of BPPV patients stratified by osteoporosis status

**Figure 1:**
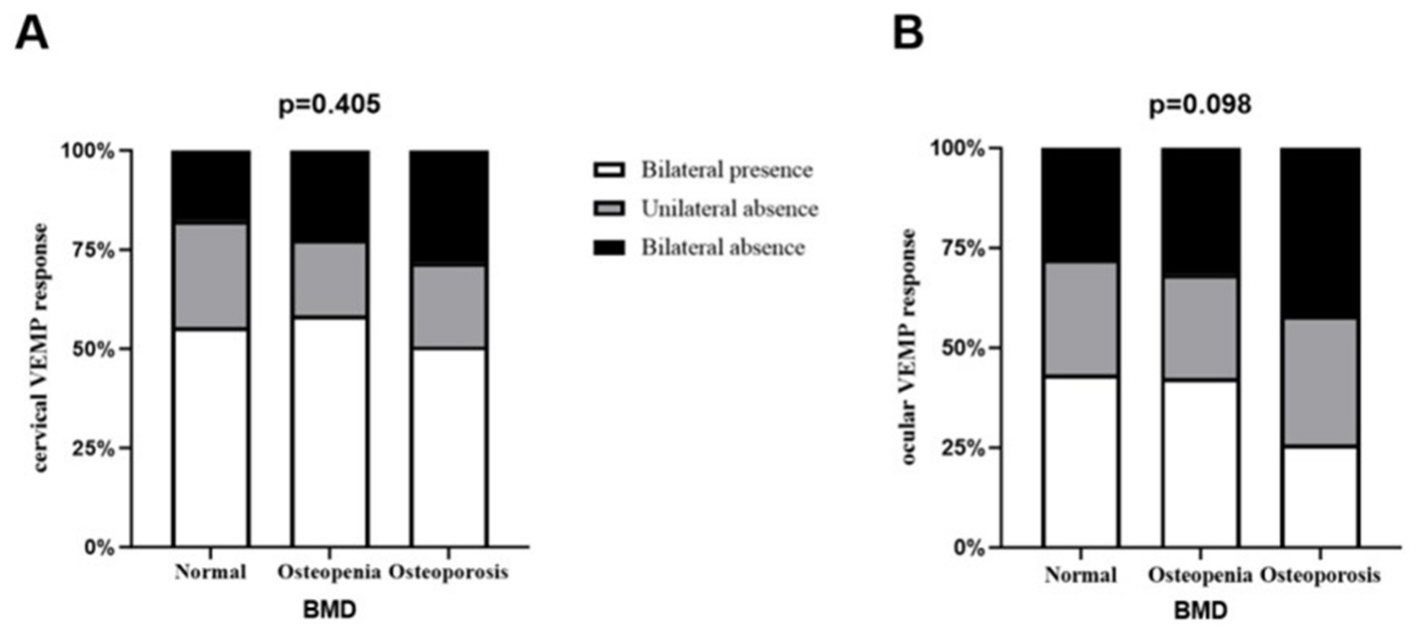
The relationship between vestibular evoked myogenic potential and bone mineral density results in benign paroxysmal positional vertigo patients.

There was a non-significant trend between BMD and oVEMP response (*p* = 0.098, Figure 1). Therefore, we divided all the participants into osteoporosis (*n* = 81) and non-osteoporosis (*n* = 210) groups for further analysis (Table 1). When compared to patients without osteoporosis, individuals with osteoporosis were typically older (65.9 ± 6.4 vs. 62.7 ± 7.2, *p* = 0.001), had decreased BMI (22.60 ± 2.71 vs. 24.29 ± 3.41, *p* < 0.001), and showed a higher proportion of females (84.0 vs. 72.4%, *p* = 0.039). No significant disparities emerged between cohorts regarding the frequency of chronic vascular diseases, duration, smoking, or drinking histories. Also, the vertigo severity at baseline and W1 was similar between the two groups. Comparative analysis of VEMP results revealed that osteoporosis patients showed significantly greater frequencies of bilateral and unilateral oVEMP absence compared to non-osteoporosis controls (*p* = 0.025). Furthermore, the absence rate of oVEMP was significantly higher in the osteoporosis group, both on the affected side (*p* = 0.036) and the unaffected side (*p* = 0.020). On the contrary, the cVEMP results were similar between the two groups. For the subjects with bilateral elicited response, no statistically significant intergroup differences emerged in any electrophysiological measures, including threshold, latency, amplitude, or AAR in patients with and without osteoporosis (Supplementary Tables 1, 2).

**Table 1:** Demographic and clinical characteristics between benign paroxysmal positional vertigo patients with and without osteoporosis.

**Variables**	**Non-osteoporosis (*n* = 210)**	**Osteoporosis (*n* = 81)**	***p*-Value**
Age, years	62.7 ± 7.2	65.9 ± 6.4	0.001^**^
BMI (kg/m^2^)	24.29 ± 3.41	22.60 ± 2.71	< 0.001^***^
Female, *n* (%)	152 (72.4%)	68 (84.0%)	0.039^*^
HBP, *n* (%)	90 (42.9%)	32 (39.5%)	0.604
DM, *n* (%)	25 (11.9%)	6 (7.4%)	0.265
HL, *n* (%)	14 (6.7%)	9 (11.1%)	0.208
CHD, *n* (%)	21 (10.0%)	10 (12.3%)	0.561
Smoking, *n* (%)	21 (10.0%)	7 (8.6%)	0.725
Drinking, *n* (%)	15 (7.1%)	2 (2.5%)	0.213
Posterior semicircular canal, *n* (%)	166 (79.0%)	63 (77.8%)	0.813
**Duration**, ***n*** **(%)**			0.545
>7 days	98 (46.7%)	41 (50.6%)	
≤ 7 days	112 (53.3%)	40 (49.4%)	
**W0**			
Vertigo VAS	90 (70–100)	90 (80–100)	0.389
Dizziness VAS	40 (10–60)	50 (20–60)	0.073
DHI total score	35.5 (20–48)	36 (24–44)	0.896
**W1**			
Vertigo VAS	0 (0–0)	0 (0–0)	0.349
Dizziness VAS	20 (0–40)	20 (0–48)	0.968
DHI total score	10 (2–22)	8 (4–22)	0.732
**The affected side**, ***n*** **(%)**			
oVEMP absence	88 (41.9%)	45 (55.6%)	0.036^*^
cVEMP absence	72 (34.3%)	32 (39.5%)	0.405
**The unaffected side**, ***n*** **(%)**			
oVEMP absence	95 (45.2%)	49 (60.5%)	0.020^*^
cVEMP absence	63 (30%)	31 (38.3%)	0.176
**oVEMP**, ***n*** **(%)**			0.025^*^
Bilateral presence	90 (42.9%)	21 (25.9%)	
Unilateral absence	57 (27.1%)	26 (32.1%)	
Bilateral absence	63 (30.0%)	34 (42.0%)	
**cVEMP**, ***n*** **(%)**			0.347
Bilateral presence	119 (56.7%)	41 (50.6%)	
Unilateral absence	47 (22.4%)	17 (21.0%)	
Bilateral absence	44 (21.0%)	23 (28.4%)	

BMI, body mass index; HBP, high blood pressure; DM, diabetes mellitus; HL, hyperlipidemia; CHD, coronary heart disease; DHI, dizziness handicap inventory; W0, at enrollment; W1, 1 week after enrollment; VAS, visual analog scale; oVEMP, ocular vestibular evoked myogenic potential; cVEMP, cervical vestibular evoked myogenic potential. ^*^p < 0.05; ^**^p < 0.01; ^***^p < 00.01.

### 3.2 Independently associated factors of BPPV patients with abnormal oVEMP

Compared with patients with bilateral oVEMP presence, those with at least unilateral absence were older (64.6 ± 7.2 vs. 62.1 ± 6.7, *p* = 0.005) and had decreased lumbar T-score and BMD values (all *p* < 0.05). Sex proportions and BMI measurements did not differ significantly groups (Table 2).

**Table 2:** Bone mineral density and T-score in the lumbar vertebrae with different ocular vestibular evoked myogenic potential response groups.

**Variables**	**Bilateral presence (*n* = 111)**	**At least unilateral absence (*n* = 180)**	***p-*Value**
Age, years	62.1 ± 6.7	64.6 ± 7.2	0.005^**^
BMI (kg/m^2^)	23.72 ± 3.33	23.88 ± 3.32	0.378
Female, *n* (%)	84 (75.7%)	136 (75.6%)	0.982
**L1**			
T-score	−1.3 ± 1.3	−1.7 ± 1.2	0.018^*^
BMD, g/cm^2^	0.896 ± 0.016	0.836 ± 0.015	0.002^**^
**L2**			
T-score	−1.3 ± 1.5	−1.7 ± 1.4	0.007^**^
BMD, g/cm^2^	0.948 ± 0.018	0.868 ± 0.017	< 0.001^***^
**L3**			
T-score	−1.1 ± 1.5	−1.7 ± 1.5	0.001^**^
BMD, g/cm^2^	0.999 ± 0.019	0.913 ± 0.018	< 0.001^***^
**L4**			
T-score	−1.0 ± 1.5	−1.4 ± 1.6	0.021^*^
BMD, g/cm^2^	0.997 ± 0.019	0.923 ± 0.019	0.002^**^
**Total**			
T-score	−1.2 ± 1.4	−1.7 ± 1.4	0.005^**^
BMD, g/cm^2^	0.964 ± 0.017	0.887 ± 0.017	< 0.001^***^

BMI, body mass index; BMD, bone mineral density; L1, the first lumbar vertebra; L2, the second lumbar vertebra; L3, the third lumbar vertebra; L4, the fourth lumbar vertebra. ^*^p < 0.05; ^**^p < 0.01; ^***^p < 0.001.

As shown in the univariate logistic regression, age (OR = 1.050, 95% CI: 1.015–1.087, *p* = 0.005) and osteoporosis (OR = 2.142, 95% CI: 1.215–3.778, *p* = 0.008) may be related to the occurrence of abnormal oVEMP, whereas sex and BMI did not emerge as contributing factors to oVEMP abnormality. After controlling the confounding factors, including age, sex, and BMI, we found that osteoporosis (OR = 2.038, 95% CI: 1.122–3.701, *p* = 0.019) was still an independent associated factor for oVEMP abnormality (Table 3).

**Table 3:** Independent risk factors for abnormal ocular vestibular evoked myogenic potential in benign paroxysmal positional vertigo patients.

**Variables**	**Univariate regression**		**Multivariate regression**	
	**OR (95% CI)**	***p*** **-Value**	**OR (95% CI)**	***p*** **-Value**
Age	1.050 (1.015–1.087)	0.005^**^	1.042 (1.006–1.080)	0.021^*^
Female	0.994 (0.573–1.724)	0.982	0.925 (0.523–1.638)	0.790
BMI	1.015 (0.944–1.091)	0.688	1.038 (0.962–1.119)	0.340
Osteoporosis	2.142 (1.215–3.778)	0.008^**^	2.038 (1.122–3.701)	0.019^*^

BMI, body mass index. ^*^p < 0.05; ^**^p < 0.01.

## 4 Discussion

For the first time, as far as we know, this study employed a cross-sectional methodology to evaluate associations linking BMD and VEMP results in elderly patients with BPPV with a large sample size. BPPV patients with osteoporosis are generally older, have a lower BMI, are more likely to be female, and have at least one unilateral absence of oVEMP. Osteoporosis is an independent factor associated with abnormal oVEMP among individuals with BPPV.

Since the utriculo-ocular pathway mediates oVEMP responses, whereas the sacculo-collic pathway underlies cVEMP responses, our study presents a novel observation: osteoporosis may be linked specifically to utricular dysfunction rather than saccular dysfunction in BPPV. Several lines of evidence support this phenomenon. (i) From a theoretical perspective, the displaced otoconia in BPPV cases primarily originate from the utricle instead of the saccule. This disorder more frequently involves utricular rather than saccular dysfunction; (ii) On a pathophysiological level, the endolymph is characterized by a low Ca2+ environment. BMD may elevate Ca2+ concentrations in the endolymph, promoting otoconia accumulation and diminishing vestibular excitation in BPPV (29, 30); (iii) In the aging population, especially in menopause women, both Baltimore Longitudinal Study of aging and a few observational studies suggested that low BMD may associate with vestibular dysfunction as evaluated by Romberg test condition 4 or VEMP tests (Table 4); (iv) A pilot study in BPPV patients in China also revealed an inverse relationship between canal paresis and low BMD (31), which also supports our results. Therefore, we speculate that osteoporosis influences calcium metabolism in the endolymph and vestibular excitation reduction, increasing the incidence of utricular dysfunction, which is involved in BPPV occurrence.

**Table 4:** Previous studies on low bone mineral density and vestibular dysfunction in humans.

**Studies**	**Subjects (sample size)**	**Evaluation methods**	**Main findings**
Mendy, et al. (21) *Ann Epidemiol*	Participants aged 40 years and older (*n* = 8,863)	BMD, Romberg test condition 4	Low BMD is associated with balance impairment, especially in older adults
Bigelow, et al. (22) *J Assoc Res Otolaryngol*	Older adults (*n* = 389)	BMD, cVEMP	Older individuals with reduced vestibular function had lower BMD
Gargeshwari, et al. (23) *Braz J Otorhinolaryngol*	Healthy controls (*n* = 12), osteopenia (*n* = 12), and osteoporosis (*n* = 11)	BMD, c/o VEMP	oVEMP (rather than cVEMP) absent rate was increased in the osteoporosis group
Juneja, et al. (24) *J Otol*	Postmenopausal women with normal BMD (*n* = 28), osteopenia (*n* = 25) and osteoporosis (*n* = 23)	BMD, c/o VEMP	The percentage of vestibular dysfunction was increased in subjects with osteopenia and osteoporosis
Jiang, et al. (30) *Otol Neurotol*	Postmenopausal women with BPPV (*n* = 65)	BMD, caloric test	CP value in the caloric test was negatively correlated with the T value in the BMD
Zhou, et al. The present study	Patients with BPPV, aged 50–80 years (*n* = 291)	BMD and c/o VEMP	Osteoporosis was an independently associated factor for at least unilateral oVEMP absence in patients with BPPV

BMD, bone mineral density; BPPV, benign paroxysmal positional vertigo; oVEMP, ocular vestibular evoked myogenic potential; cVEMP, cervical vestibular evoked myogenic potential; CP, canal paresis.

Gargeshwari et al. (23) reported a significantly higher rate of oVEMP absence in individuals with osteoporosis compared to healthy controls; however, no statistically significant differences were observed in the cVEMP or oVEMP parameters between the two groups, which is consistent with our findings. Another study found no significant difference in the absence rate or parameters of cVEMP between individuals with vitamin D deficiency and healthy control (32). This suggests that the saccule may exhibit reduced sensitivity to alterations in calcium metabolism. In contrast, impaired bone metabolism is more likely to lead to utricular dysfunction, which may manifest as abnormal oVEMP findings. In other vestibular disorders, objective test findings do not necessarily worsen in parallel. For example, in Ménière's disease, cochlear and vestibular impairments do not show a clear correlation. In our study, osteoporosis was primarily associated with absent oVEMP responses rather than cVEMP, suggesting an otolith-organ–specific effect. This finding is consistent with the hypothesis that otoconia dislodge from the utricle in BPPV (33).

Our results demonstrate that osteoporosis exerts a bilateral effect on oVEMP. This aligns with previous findings in patients with diabetic peripheral neuropathy, who also exhibited significantly prolonged c/o VEMP latencies without notable interaural differences (34). We therefore propose that systemic metabolic disturbances in osteoporosis induce bilateral alterations in the endolymph and otolithic structures, providing a mechanistic explanation for the clinically observed unpredictability of the affected side in BPPV patients.

Our investigation demonstrated that participants in the osteoporosis group, as well as those exhibiting abnormal oVEMP, were generally older, aligning with previous animal studies. Both bone metabolism and vestibular system function decline with age. Existing literature indicates that vestibular balance in older mice is more susceptible to impairment compared to younger mice, and cholinergic receptor transmission within the vestibular hair cells is less efficient in aged mice than in their younger counterparts (35, 36).

Notably, the majority of our study participants were older women (75.6%). Previous studies have shown that around the age of 50, women gradually undergo menopause, during which estrogen levels begin to decline rapidly, leading to a diminished inhibitory effect on osteoclasts and resulting in osteoporosis (37). Moreover, Estrogen deficiency disrupts otoconia stability and attachment by downregulating proteins involved in otoconial formation and anchoring (38). Thus, hormonal factors may confound the observed association between BMD and oVEMP abnormalities. However, osteoporosis remained significantly associated with oVEMP abnormalities after adjusting for age and sex in multivariable models, suggesting that the bone-vestibular link is not solely explained by hormonal status, and future studies should incorporate these variables.

Our study has important clinical implications for linking osteoporosis with utricular dysfunction in BPPV, which contributes to disease occurrence. From a clinical perspective, our findings highlight the importance of routine BMD screening in older subjects presenting with BPPV. Moreover, given that osteoporotic BPPV patients are more likely to exhibit oVEMP abnormalities, anti-osteoporotic treatment may be indicated for this subgroup to improve the disease prognosis. A few limitations should be mentioned. We did not include healthy controls or osteoporotic individuals without BPPV. Future studies should include age- and sex-matched control groups to determine whether the observed oVEMP abnormality is specific to BPPV. Also, vestibular function was solely evaluated by VEMPs, caloric tests and video-head impulse tests (v-HITs) were not performed, which meant that semicircular canal function and high-frequency vestibular-ocular reflex integrity were not assessed. Meanwhile, our vestibular testing was performed within 1 week after successful CRP, residual dizziness, transient vestibular hypofunction, or incomplete central compensation may influence the VEMP results (39). More detailed vestibular evaluations at different time point after CRPs with a multicenter design are needed in the future.

In summary, our study provides further evidence suggesting that osteoporosis may play a role in utricular dysfunction associated with the development of BPPV. The potential for osteoporosis treatment to improve disease prognosis in patients with BPPV warrants additional research.

## Data Availability

The raw data supporting the conclusions of this article will be made available by the authors, without undue reservation.
