# Supplementary material for: Osteoporosis correlates with abnormal ocular vestibular evoked myogenic potential in patients with benign paroxysmal positional vertigo
Source: Front Neurol. 2026 Mar 12;17:1785323. doi: 10.3389/fneur.2026.1785323 (PMC13017236; doi:10.3389/fneur.2026.1785323)
Supplement: Supplementary file 1 [file Table_1.docx]

Supplementary Material

# Supplementary Tables

Supplementary Table 1. The specific characteristics of ocular vestibular evoked myogenic potential in the benign paroxysmal positional vertigo patients with bilateral elicited response

| **Variable** | **Non-osteoporosis(*n*=85)** | **Osteoporosis (*n*=19)** | ***p* value** |
| --- | --- | --- | --- |
| **Right oVEMP** |  |  |  |
| Threshold (dBnHL) | 105(100-110) | 105(100-110) | 0.609 |
| N10 latency (ms) | 12.5±1.0 | 11.2±1.2 | 0.591 |
| P15 latency (ms) | 16.6±1.4 | 16.2±1.3 | 0.310 |
| Amplitude (μV) | 3.8(2.9-4.9) | 4.6(3.1-6.2) | 0.222 |
| **Left oVEMP** |  |  |  |
| Threshold (dBnHL) | 105(100-110) | 105(100-110) | 0.285 |
| N10 latency (ms) | 12.4±0.9 | 11.2±1.2 | 0.614 |
| P15 latency (ms) | 16.4±1.4 | 16.2±1.9 | 0.551 |
| Amplitude (μV) | 5.3(3.6-7.6) | 4.2(3.2-6.2) | 0.252 |
| **AAR, *n* (%)** |  |  | 0.068 |
| ≥25% | 15(17.6%) | 6(31.6%) |  |
| ≤-25% | 10(11.8%) | 0(0%) |  |
| -25%～25% | 60(70.6%) | 13(68.4%) |  |

Notes: AAR, amplitude asymmetry ratio. AAR= 100×[Ar−Al]/[Ar+Al] where Ar was the amplitude of the right ear and Al was that of the left ear.**p* < 0.05; ***p* < 0.01; ****p* < 0.001

Supplementary Table 2. The specific characteristics of cervical vestibular evoked myogenic potential in the benign paroxysmal positional vertigo patients with bilateral elicited response

| **Variable** | **Non-osteoporosis (*n*=107)** | **Osteoporosis (*n*=37)** | ***p* value** |
| --- | --- | --- | --- |
| **Right cVEMP** |  |  |  |
| Threshold (dBnHL) | 100(95-105) | 100(100-105) | 0.485 |
| P13 latency (ms) | 14.2±1.4 | 13.9±1.2 | 0.179 |
| N23 latency (ms) | 22.6±2.0 | 22.2±1.7 | 0.276 |
| Amplitude (μV) | 48.7(37.5-71.9) | 54.4(39.9-74.5) | 0.446 |
| **Left cVEMP** |  |  |  |
| Threshold (dBnHL) | 105(100-110) | 100(95-110) | 0.517 |
| P13 latency (ms) | 14.6±1.6 | 14.6±2.0 | 0.570 |
| N23 latency (ms) | 22.7±2.0 | 22.5±1.5 | 0.983 |
| Amplitude (μV) | 48.7(37.5-83.8) | 49.6(38.4-65. 1) | 0.507 |
| **AAR, *n* (%)** |  |  | 0.135 |
| ≥25% | 12(11.2%) | 9(24.3%) |  |
| ≤-25% | 21(19.6%) | 5(13.5%) |  |
| -25%～25% | 74(69.2%) | 23(62.2%) |  |

Notes: AAR, amplitude asymmetry ratio. AAR= 100× (right-side amplitude - left-side amplitude) / (right-side amplitude + left-side amplitude).**p* < 0.05; ***p* < 0.01; ****p* < 0.001
